# Transgenerational Epigenetic Contributions to Stress Responses: Fact or Fiction?

**DOI:** 10.1371/journal.pbio.1002426

**Published:** 2016-03-25

**Authors:** Eric J. Nestler

**Affiliations:** Fishberg Department of Neuroscience and Friedman Brain Institute, Icahn School of Medicine at Mount Sinai, New York, New York, United States of America

## Abstract

There has been increasing interest in the possibility that behavioral experience—in particular, exposure to stress—can be passed on to subsequent generations through heritable epigenetic modifications. The possibility remains highly controversial, however, reflecting the lack of standardized definitions of epigenetics and the limited empirical support for potential mechanisms of transgenerational epigenetic inheritance. Nonetheless, growing evidence supports a role for epigenetic regulation as a key mechanism underlying lifelong regulation of gene expression that mediates stress vulnerability. This Perspective provides an overview of the multiple meanings of the term epigenetic, discusses the challenges of studying epigenetic contributions to stress susceptibility—and the experimental evidence for and against the existence of such mechanisms—and outlines steps required for future investigations.

## Abstract 2

Can behavioral experience—and, in particular, exposure to stress—be passed on to subsequent generations via heritable epigenetic modifications? This Perspective assesses the evidence for and against.

## Introduction

The term epigenetics is used in at least three different ways, each referring to a fundamentally different mode of biological regulation, which has contributed to considerable confusion in the field. Epigenetics, in its broadest meaning, is used by some to denote stable changes in gene expression that are mediated via mechanisms that do not involve modification of DNA sequence. In the field of stress responses, such epigenetically induced stable changes in gene expression likely result from any number of environmental stresses that occur throughout a lifetime. For example, chronic stress may induce epigenetic mechanisms that alter gene expression in the adult brain [1]. Likewise, it’s thought that environmental exposures to stress and other behavioral experience early in life (i.e., in utero and during childhood and adolescence) may produce epigenetic changes that determine how susceptible or resilient an individual is to those or other stresses later in life. There is now robust and growing evidence supporting a role for epigenetic modification as a key mechanism underlying lifelong regulation of gene expression and, consequently, of stress vulnerability [2–12].

The term epigenetic is also used to describe two additional phenomena for which the evidence base is less solid. One concerns stochastic changes during development, whereby random epigenetic modifications in the developing brain generate variations in an individual’s traits, including differences in stress vulnerability, without changes in either genomic sequence or environmental exposures. The other relates to epigenetic inheritance across multiple generations, whereby epigenetic modifications induced in an individual’s germ cells in response to stress or other environmental exposures are transmitted to offspring to control their stress vulnerability. The degree to which these two forms of epigenetic regulation contribute to the lasting consequences of stress, including stress-related syndromes such as depression, remains more controversial.

## Brief Overview of Epigenetic Mechanisms

**Fig 1:**
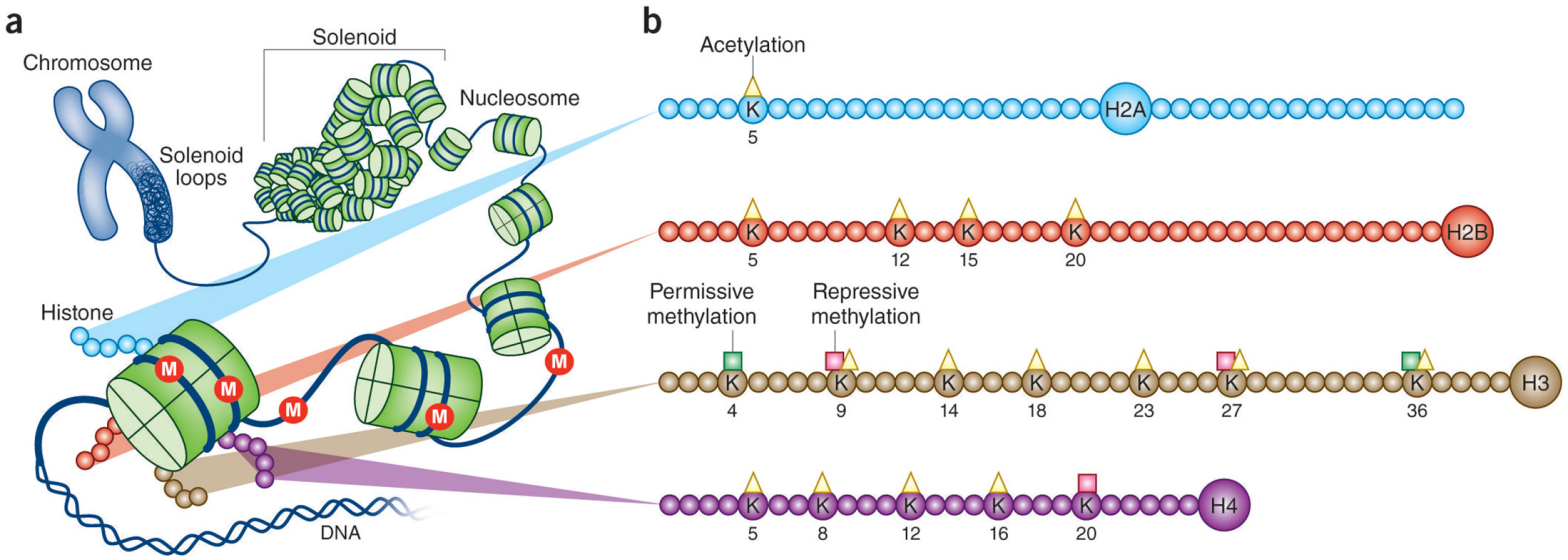
Chromatin structure and histone modifications at N-terminal histone tails. (A) The eukaryotic genome is organized by wrapping DNA around histone octamers to form the basic units of chromatin, nucleosomes, which are then further organized and compacted into higher ordered structures. (B) The histone octamer consists of two copies each of H2A, H2B, H3, and H4. In addition to globular domains, they each have N-termini tails that protrude from the nucleosome, while H2A also has a C-terminal tail. These tails can be post-translationally modified, and major forms of mammalian acetylation and methylation modifications on lysine residues on each tail are highlighted. The molecules are drawn roughly to proportion to the size of the protein, although the number of residues shown is not meant to reflect the exact size of the N-terminal tails. In addition to histone modifications, DNA itself is modified by methylation or hydroxymethylation (M), among other alterations. Adapted from [15].

DNA is wrapped around histone octomers to form nucleosomes—the unit of chromatin (Fig 1). A gene’s activity is reflected in the surrounding structure of chromatin: genes within relatively spaced nucleosomes are actively transcribed, whereas those in tightly packed nucleosomes are silenced. Such nucleosome spacing is determined by extremely complex processes, which include the post-translational modification of histones (see Fig 1) and DNA as well as the recruitment of large numbers of chromatin regulatory proteins that interact with these modifications [13,14]. On the other hand, there continues to be disagreement, partly semantic, about whether alterations in epigenetic states per se cause changes in gene expression or whether they reflect changes mediated by other mechanisms.

Post-translational modifications of histones remain the best studied form of epigenetic regulation. Each of the four canonical histone subunits in mammals is modified at specific amino acid residues in diverse ways, including acetylation, methylation, polyADP-ribosylation, and ubiquitination, among many others. DNA is also modified, without affecting nucleotide sequence, most prominently through its methylation, hydroxymethylation, or related changes (Fig 1). Meanwhile, large numbers of regulatory proteins bind to each of these histone and DNA modifications and mediate changes in chromatin structure and gene transcription. As just one example, chromatin remodeling proteins possessing ATPase activity control nucleosome spacing and positioning during transcription. Work over recent years has also demonstrated several forms of noncoding RNAs, including microRNAs (miRNAs) and long noncoding RNAs (lncRNAs), as important mediators of epigenetic regulation. There is now growing evidence, alluded to above, that each of these forms of epigenetic regulation are altered in the brain by exposure to stress and that their experimental manipulation (e.g., by overexpression or knockout of a chromatin regulatory protein or delivery of small molecule inhibitors) has dramatic effects on stress vulnerability [2–12].

Environmental stimuli, such as stress exposures over a lifetime, regulate these various epigenetic processes in two main ways [16]. First, synaptic transmission and neural activity, through intracellular signaling cascades, control the activity and levels of numerous transcription factors—e.g., CREB (cAMP response element binding protein) and Fos and Jun family proteins, among many others—that bind to their specific response elements within regulatory regions of genes and trigger downstream changes in chromatin structure. Second, the same intracellular signaling pathways directly control the activity or expression of many chromatin regulatory proteins (e.g., histone- or DNA-modifying enzymes, chromatin remodeling factors), which then directly drive alterations in gene expression. Indeed, a recent study established that single types of histone modifications at a single gene within neurons in an adult brain, induced through the viral-mediated expression of synthetic transcription factors, is sufficient to induce altered expression of that gene, thus establishing this second pathway of gene regulation [17].

## Stochastic Epigenetic Contributions to Depression

As the human brain develops, generating ~100 billion neurons and ~100 trillion synapses, enumerable stochastic events occur that generate diversity even though genetics and environment remain constant [18,19]. The highly divergent patterns of cerebral gyri exhibited by identical twins is an example of such phenomena (identical twins also have different fingerprints, another example of presumably random events during development). While epigenetic mechanisms are likely contributors to such differences, it is difficult to obtain experimental proof. For example, in the chronic social defeat stress model of depression, genetically identical animals with virtually uniform environmental exposures diverge strikingly into susceptible versus resilient outcomes after social stress [20]. However, it is not currently possible to define epigenetic contributions to such phenotypic outcomes, because it is not possible at the present time to determine—prior to stress exposure—which individuals will show susceptibility or resilience, making it impossible to characterize the preexisting epigenetic state of genes within animals prior to stress.

An alternative approach would be to compare stress-induced changes in epigenetic state in a given brain region or cell type of a susceptible versus resilient adult mouse after social defeat stress to the epigenetic states seen in the same brain region or cell type of mice with or without exposure to stress or other challenges early in life. In fact, preliminary evidence suggests that a form of early life stress, which renders a mouse more susceptible to social defeat stress later in life, induces patterns of gene regulation in several brain regions—in the absence of adult stress—that resemble patterns induced by adult stress in susceptible mice [21]. However, this is a fundamentally different question from the hypothesized role of stochastic events during development. In the latter case, two mice from the same litter with identical environmental exposures would develop different epigenetic states in the brain, due to no extrinsic factors—just random events during development, which make one of the mice susceptible after social defeat and the other resilient, even though we cannot distinguish the mice behaviorally before social defeat.

In any event, stochastic, epigenetic differences in brain function could be one reason why it has been difficult to identify genes that confer risk for depression and other stress-related disorders. This use of the term epigenetics comes closest to Waddington’s original definition in 1942, which posited—before we knew that DNA carried genetic information—that the effect of genetics in determining phenotype is subject to random variations during development, akin to several marbles taking distinct paths rolling down a hill [22].

## Epigenetic Inheritance of Depression Susceptibility

The term epigenetics is also used to refer to the transgenerational transmission of traits without a change in DNA sequence [23,24]. It is clear that stressful life events can alter stress susceptibility in subsequent generations. Male mouse pups subjected to maternal separation display lifelong increases in stress susceptibility and generate offspring that display similarly enhanced stress susceptibility over several generations [25]. Adult male mice subjected to chronic social defeat stress generate offspring that are more vulnerable to a range of stressful stimuli than the offspring of control mice [26]. Likewise, adolescent or adult male mice subjected to chronic variable stress sire offspring that display aberrant stress regulation of the hypothalamic-pituitary-adrenal axis as well as of gene expression in stress-sensitive brain regions [27].

However, the mechanisms underlying this clear transgenerational transmission of stress vulnerability remain controversial. Stress produces DNA methylation changes at particular genes in the sperm of stressed mice [25], yet it is far from certain whether such modifications contribute to the differences seen in stress vulnerability (see below). Studies utilizing in vitro fertilization—which has its own confounds (see below)—after chronic social defeat stress suggest that, while epigenetic changes in sperm might be a small factor in transgenerational transmission of stress vulnerability, a large portion of the observed transmission may be behavioral, with females altering their maternal care based on their procreation with previously stressed fathers [26].

Such skepticism for the transgenerational epigenetic inheritance of stress susceptibility is based on current schemes whereby virtually all epigenetic modifications that occur throughout life are “erased” during meiosis [28,29]. Even imprinted genomic loci—those displaying increased DNA methylation selectively on maternal versus paternal alleles—are erased during meiosis and then recapitulated later in development. Nevertheless, there appear to be a small number of genomic regions that might not be subjected to demethylation, such that this possible mechanism of transgenerational epigenetic inheritance warrants future attention. Likewise, evidence from nonvertebrate systems suggests that certain types of histone modifications may play a role in mediating transgenerational epigenetic inheritance [23].

Additionally, increasing attention has been given to miRNAs as possible vehicles of transgeneration epigenetic inheritance. In worms, for example, phenotypes as varied as viral immunity, nutritional status, and aging can be influenced across several generations via induction of specific miRNAs in parents [30–32]. Growing evidence implicates similar mechanisms in transgenerational control of stress responses in mice. Both maternal separation early in life and chronic variable stress later in adulthood alter levels of several miRNAs in sperm [27,33]. Moreover, injection of total sperm RNA [33] or injection of nine stress-regulated miRNAs [34] into normal zygotes recapitulates the transgenerational transmission of behavioral, hormonal, and gene expression deficits in offspring animals. Such recent studies are, thus, getting much closer to providing definitive evidence for true epigenetic inheritance of stress susceptibility, although it would be important to knockout or recapitulate changes in specific miRNAs in sperm and show how they subsequently influence the brains of offspring animals to alter stress responses.

The failure of in vitro fertilization to demonstrate robust epigenetic inheritance of social defeat-induced susceptibility (stated above) [26] may not be surprising, since substantial epigenetic reprogramming occurs when sperm or egg cells are placed in vitro. In fact, a more recent study, in which normal females have sex with normal (but vasectomized) males, followed immediately by artificial insemination with sperm from defeated or control fathers, supports that sperm can indeed be an active vehicle in transmitting increased stress susceptibility to the next generation [35]. It will be interesting in future studies to determine if this effect, too, is mediated by miRNAs.

The final consideration in studying transgenerational epigenetic inheritance is the fact that most groups have examined paternal transmission. This is due to two practical considerations. First, studying paternal transmission is one generation easier, since the eggs of the F1 generation are present in mothers exposed to stress or any other stimulus, and the eggs of the F2 generation are present in those F1 pups. As a result, the first generation that could show true transgenerational inheritance is F3. Second, it is well established that different amounts or types of maternal care can have a profound, lasting effect on stress-related behavior in offspring animals [8,36], which makes it that much harder to parse the effects of epigenetic mechanisms versus maternal behavior when studying maternal transmission of traits. Despite these challenges, it is essential to evaluate epigenetic inheritance by maternal as well as paternal transmission.

## Conclusions

Further work is needed to understand whether and to what extent true epigenetic inheritance of stress vulnerability adds to the well-established and powerful influence of genetics and environmental exposures in determining an individual’s susceptibility versus resilience to stress throughout life. Despite the two extremes of disbelief versus wild speculation, there is growing evidence for at least some contribution of epigenetic regulation—perhaps achieved by miRNAs—in mediating part of the ability of parental behavioral experience to influence stress vulnerability in their offspring.

## Abbreviations

CREB: cAMP response element binding protein
lncRNA: long noncoding RNAs
miRNA: microRNA

## References

[1] Vialou V, Feng J, Robison AJ, Nestler EJ. Epigenetic mechanisms of depression and antidepressant action. Annu Rev Pharmacol Exp Ther. 2012;53:59–87 10.1146/annurev-pharmtox-010611-134540 PMC3711377 23020296

[2] Covington HE 3rd, Maze I, LaPlant QC, et al Antidepressant actions of histone deacetylase inhibitors. J Neurosci. 2009;29:11451–11460. 10.1523/JNEUROSCI.1758-09.2009 19759294 PMC2775805

[3] Covington HE 3rd, Maze I, Sun H, et al A role for repressive histone methylation in cocaine-induced vulnerability to stress. Neuron. 2011;71:656–670. 10.1016/j.neuron.2011.06.007 21867882 PMC3163060

[4] Hunter RG, McCarthy KJ, Milne TA, Pfaff DW, McEwen BS. Regulation of hippocampal H3 histone methylation by acute and chronic stress. Proc Natl Acad Sci U S A. 2009;106:20912–20917. 10.1073/pnas.0911143106 19934035 PMC2791599

[5] Tsankova NM, Berton O, Renthal W, Kumar A, Neve RL, Nestler EJ. Sustained hippocampal chromatin regulation in a mouse model of depression and antidepressant action. Nat Neurosci. 2006;9:519–525. 16501568 10.1038/nn1659

[6] Wilkinson MB, Xiao GH, Kumar A, et al Imipramine treatment and resiliency exhibit similar chromatin regulation in a key brain reward region. J Neurosci. 2009;29:7820–7832. 10.1523/JNEUROSCI.0932-09.2009 19535594 PMC2717944

[7] Uchida S, Hara K, Kobayashi A, et al Epigenetic status of Gdnf in the ventral striatum determines susceptibility and adaptation to daily stressful events. Neuron. 2011;69:359–372. 10.1016/j.neuron.2010.12.023 21262472

[8] Weaver IC, Cervoni N, Champagne FA, et al Epigenetic programming by maternal behavior. Nat Neurosci. 2014;7:847–854. 10.1038/nn1276 15220929

[9] Jiang Y, Jakovcevski M, Bharadwaj R, et al Setdb1 histone methyltransferase regulates mood-related behaviors and expression of the NMDA receptor subunit NR2B. J Neurosci. 2010;30:7152–7167. 10.1523/JNEUROSCI.1314-10.2010 20505083 PMC2893142

[10] LaPlant Q, Vialou V, Covington HE 3rd, et al Dnmt3a regulates emotional behavior and spine plasticity in the nucleus accumbens. Nat Neurosci. 2010;13:1137–1143. 10.1038/nn.2619 20729844 PMC2928863

[11] Dias C, Feng J, Sun HS, et al β-Catenin mediates stress resilience through Dicer1/microRNA regulation. Nature. 2014;516:51–55. 10.1038/nature13976 25383518 PMC4257892

[12] Sun HS, Damez-Werno D, Scobie KN, et al ACF chromatin remodeling complex mediates stress-induced depressive-like behavior. Nat. Med. 2015;21:1146–1153. 10.1038/nm.3939 26390241 PMC4598281

[13] Maze I, Noh KM, Allis CD. Histone regulation in the CNS: basic principles of epigenetic plasticity. Neuropsychopharmacology. 2013;38:3–22. 10.1038/npp.2012.124 22828751 PMC3521967

[14] Suzuki MM, Bird A. DNAmethylation landscapes: provocative insights from epigenomics. Nat Rev Genet. 2008;9:465–76. 10.1038/nrg2341 18463664

[15] Sun HS, Kennedy PJ, Nestler EJ. Epigenetics of the depressed brain: Role of histone acetylation and methylation. Neuropsychopharmacology Rev. 2013;38:124–137. 10.1038/npp.2012.73 PMC3521990 22692567

[16] Robison AJ, Nestler EJ. Transcriptional and epigenetic mechanisms of addiction. Nat Rev Neurosci. 2011;12:623–637. 10.1038/nrn3111 21989194 PMC3272277

[17] Heller EA, Cates HM, Peña CJ, et al Locus-specific epigenetic remodeling controls addiction- and depression-related behaviors. Nat Neurosci. 2014;17:1720–1727. 10.1038/nn.3871 25347353 PMC4241193

[18] Czyz W, Morahan JM, Ebers GC, Ramagopalan V. Genetic, environmental and stochastic factors in monozygotic twin discordance with a focus on epigenetic differences. BMC Med. 2012;10:93 10.1186/1741-7015-10-93 22898292 PMC3566971

[19] Feinberg AP, Irizarry RA. Stochastic epigenetic variation as a driving force of development, evolutionary adaptation, and disease. Proc Natl Acad Sci USA. 2010;107(suppl 1):1757–1764. 10.1073/pnas.0906183107 20080672 PMC2868296

[20] Krishnan V, Han MH, Graham DL, et al Molecular adaptations underlying susceptibility and resistance to social defeat in brain reward regions. Cell. 2007;131:391–404. 17956738 10.1016/j.cell.2007.09.018

[21] Pena CJ, Purushothaman I, Cates HM, Bagot RC, Walker DM, Shen L, Nestler EJ. Early life stress enhances susceptibility to depression via long-lasting transcriptional alterations. Soc Neurosci Abs. 2015;375.04.

[22] Waddington CH. The epigenotype. 1942. Int J Epidemiol. 2012;41:10–13. 10.1093/ije/dyr184 22186258

[23] Lim JP, Brunet A. Bridging the transgenerational gap with epigenetic memory. Trends Genet. 2013;29:176–186. 10.1016/j.tig.2012.12.008 23410786 PMC3595609

[24] Bale TL. Lifetime stress experience: transgenerational epigenetics and germ cell programming. Dialogues Clin Neurosci. 2014;16:297–305. 25364281 10.31887/DCNS.2014.16.3/tbale PMC4214173

[25] Franklin TB, Russig H, Weiss IC, et al Epigenetic transmission of the impact of early stress across generations. Biol Psychiatry. 2010;68:408–415. 10.1016/j.biopsych.2010.05.036 20673872

[26] Dietz DM, LaPlant Q, Watts EL, et al Paternal transmission of stress-induced pathologies. Biol Psychiatry. 2011;70:408–414. 10.1016/j.biopsych.2011.05.005 21679926 PMC3217197

[27] Rodgers AB, Morgan CP, Bronson SL, Revello S, Bale TL. Paternal stress exposure alters sperm microRNA content and reprograms offspring HPA stress axis regulation. J Neurosci. 2013;33:9003–9012. 10.1523/JNEUROSCI.0914-13.2013 23699511 PMC3712504

[28] Cantone I, Fisher AG. Epigenetic programming and reprogramming during development. Nat Struct Mol Biol. 2013;20: 282–289. 10.1038/nsmb.2489 23463313

[29] von Meyenn F, Reik W. Forget the parents: Epigenetic reprogramming in human germ cells. Cell. 2015;161:1248–1251. 10.1016/j.cell.2015.05.039 26046435

[30] Greer EL, Maures TJ, Ucar D, et al Transgenerational epigenetic inheritance of longevity in Caenorhabditis elegans. Nature. 2011;479:365–371. 10.1038/nature10572 22012258 PMC3368121

[31] Rechavi O, Houri-Ze'evi L, Anava S, et al Starvation-induced transgenerational inheritance of small RNAs in C. elegans. Cell. 2014;158:277–287. 10.1016/j.cell.2014.06.020 25018105 PMC4377509

[32] Rechavi O, Minevich G, Hobert O. Transgenerational inheritance of an acquired small RNA-based antiviral response in C. elegans. Cell. 2011;147:1248–1256. 10.1016/j.cell.2011.10.042 22119442 PMC3250924

[33] Gapp K, Jawaid A, Sarkies P, Bohacek J, Pelczar P, Prados J, Farinelli L, Miska E, Mansuy IM. Implication of sperm RNAs in transgenerational inheritance of the effects of early trauma in mice. Nat Neurosci. 2014;17:667–669. 10.1038/nn.3695 24728267 PMC4333222

[34] Rodgers AL, Morgan CP, Leu NA, Bale TL. Transgenerational epigenetic programming via sperm microRNA recapitulates effects of paternal stress. Proc Natl Acad Sci USA. 2015;112:13699–13704. 10.1073/pnas.1508347112 26483456 PMC4640733

[35] Walker DM, Doyle MA, Bagot RC, et al Paternal transmission of stress-induced phenotypes are transmitted via male germ cells. Soc Neurosci Abs. 2015;504.03.

[36] Zhang TY, Labonté B, Wen XL, Turecki G, Meaney MJ. Epigenetic mechanisms for the early environmental regulation of hippocampal glucocorticoid receptor gene expression in rodents and humans. Neuropsychopharmacology. 2013;38:111–123. 10.1038/npp.2012.149 22968814 PMC3521971

